# SVM and SVM Ensembles in Breast Cancer Prediction

**DOI:** 10.1371/journal.pone.0161501

**Published:** 2017-01-06

**Authors:** Min-Wei Huang, Chih-Wen Chen, Wei-Chao Lin, Shih-Wen Ke, Chih-Fong Tsai

**Affiliations:** 1 Department of Psychiatry, Chiayi Branch, Taichung Veterans General Hospital, Chiayi, Taiwan; 2 Department of Pharmacy, Kaohsiung Municipal Chinese Medical Hospital, Kaohsiung, Taiwan; 3 Graduate Institute of Natural Products, Kaohsiung Medical University, Kaohsiung, Taiwan; 4 Department of Computer Science and Information Engineering, Asia University, Taichung, Taiwan; 5 Department of Information and Computer Engineering, Chung Yuan Christian University, Taoyuan, Taiwan; 6 Department of Information Management, National Central University, Taoyuan, Taiwan; Instituto Nacional de Medicina Genomica, MEXICO

## Abstract

Breast cancer is an all too common disease in women, making how to effectively predict it an active research problem. A number of statistical and machine learning techniques have been employed to develop various breast cancer prediction models. Among them, support vector machines (SVM) have been shown to outperform many related techniques. To construct the SVM classifier, it is first necessary to decide the kernel function, and different kernel functions can result in different prediction performance. However, there have been very few studies focused on examining the prediction performances of SVM based on different kernel functions. Moreover, it is unknown whether SVM classifier ensembles which have been proposed to improve the performance of single classifiers can outperform single SVM classifiers in terms of breast cancer prediction. Therefore, the aim of this paper is to fully assess the prediction performance of SVM and SVM ensembles over small and large scale breast cancer datasets. The classification accuracy, ROC, F-measure, and computational times of training SVM and SVM ensembles are compared. The experimental results show that linear kernel based SVM ensembles based on the bagging method and RBF kernel based SVM ensembles with the boosting method can be the better choices for a small scale dataset, where feature selection should be performed in the data pre-processing stage. For a large scale dataset, RBF kernel based SVM ensembles based on boosting perform better than the other classifiers.

## Introduction

Breast cancer prediction has long been regarded as an important research problem in the medical and healthcare communities. This cancer develops in the breast tissue [1]. There are several risk factors for breast cancer including female sex, obesity, lack of physical exercise, drinking alcohol, hormone replacement therapy during menopause, ionizing radiation, early age at first menstruation, having children late or not at all, and older age.

There are different types of breast cancer, with different stages or spread, aggressiveness, and genetic makeup. Therefore, it would be very useful to have a system that would allow early detection and prevention which would increase the survival rates for breast cancer.

The literature discusses a number of different statistical and machine learning techniques that have been applied to develop breast cancer prediction models, such as logistic regression, linear discriminate analysis, naïve Bayes, decision trees, artificial neural networks, k-nearest neighbor, and support vector machine methods [2–9].

More specifically, studies comparing some of the above mentioned techniques have shown that SVM performs better than many of the other related techniques [10–15].

It is necessary when constructing the SVM classifier to determine a specific kernel function, such as a polynomial or radial basis function (RBF), which is an important learning parameter. However, there have been very few studies focused on assessing the prediction performance of SVM classifiers constructed using different kernel functions. In addition, it is known that combining multiple classifiers or classifier ensembles, another active research area of pattern classification, often gives better performance than single classifiers [16]. However, with the exception of Lavanya and Rani [17] who show that decision tree ensembles constructed by bagging perform better than the single decision tree model, the performance of classifier ensembles in breast cancer prediction has not often been explored. Thus, it is unknown whether SVM ensembles can outperform single SVM classifiers in breast cancer prediction.

Another complicating factor is that the collected dataset for breast cancer prediction is usually class imbalanced, with the minority class containing a small number of patients with cancer and the majority class containing a large number of patients without cancer. This means that using only prediction accuracy or classification accuracy to evaluate the prediction models is insufficient [18]. Other evaluation metrics that use different types of classification errors, such as the area under the curve (AUC) or the receiver operating characteristic (ROC) curve [19], should also be examined to fully understand the performance of the prediction model.

Therefore, our research objective is to compare SVM and SVM ensembles using different kernel functions (i.e., linear, polynomial, and RBF kernel functions) and combination methods (i.e., bagging and boosting). Their performance will be assessed by different evaluation metrics, including the classification accuracy, ROC, F-measure, and classifier training time. Consequently, the findings of this paper should allow future researchers to easily choose the most effective baseline technique that can provide the optimal prediction performance for future comparison.

The rest of this paper is organized as follows. Section 2 overviews related studies including those on support vector machines and classifier ensembles, and compares related works in terms of the kernel function employed, the dataset used, and the evaluation metric considered. Section 3 describes the experimental methodology including the experimental procedure, the imputation process and experimental setup. Section 4 presents the experimental results. Finally, Section 5 concludes the paper.

## Literature Review

### Support Vector Machines

Support vector machines (SVMs), first introduced by Vapnik [20], have shown their effectiveness in many pattern recognition problems [21], and they can provide better classification performances than many other classification techniques.

An SVM classifier performs binary classification, i.e., it separates a set of training vectors for two different classes (*x*_*1*_, *y*_*1*_), (*x*_*2*_, *y*_*2*_),…, (*x*_*m*_, *y*_*m*_), where *x*_*i*_ ∈ *R*^*d*^ denotes vectors in a *d*-dimensional feature space and *y*_*i*_ ∈ {-1, +1} is a class label. The SVM model is generated by mapping the input vectors onto a new higher dimensional feature space denoted as Φ: *R*^*d*^ → *H*^*f*^ where *d* < *f*. Then, an optimal separating hyperplane in the new feature space is constructed by a kernel function *K*(*x*_*i*_,*x*_*j*_), which is the product of input vectors *x*_*i*_ and *x*_*j*_ and where *K*(*x*_*i*_,*x*_*j*_) = Φ(*x*_*i*_) · Φ(*x*_*j*_).

**Fig 1:**
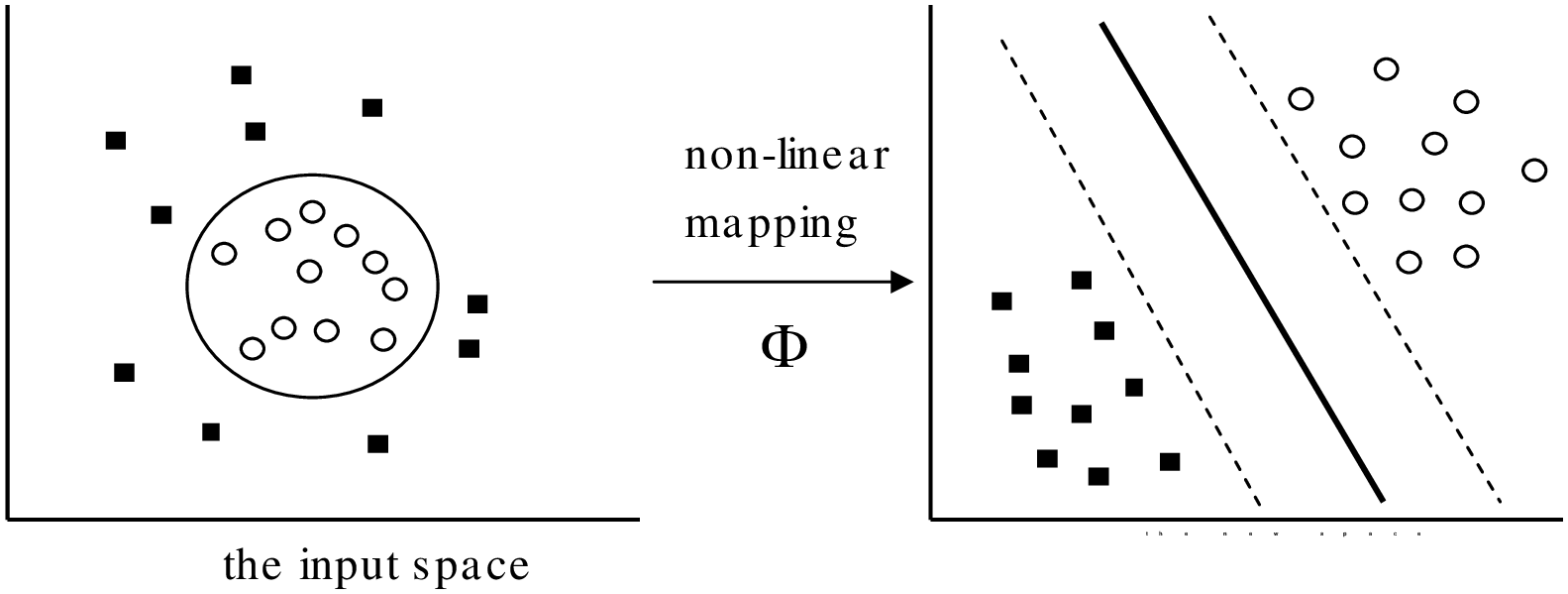
SVM model generation.

Fig 1 illustrates this procedure of a linear kernel based SVM, which maps the nonlinear input space into the new linearly separable space. In particular, all vectors lying on one side of the hyperplane are labelled as -1, and all vectors lying on another side are labelled as +1. The training instances that lie closest to the hyperplane in the transformed space are called support vectors. The number of these support vectors is usually small compared to the size of the training set and they determine the margin of the hyperplane, and thus the decision surface.

Two widely used kernel functions are the Polynomial and Gaussian Radial Basis Function (RBF) kernel functions, which are *K*_*poly*_(*x*_*i*_,*x*_*j*_) = (*x*_*i*_ · *x*_*j*_ + 1)^*p*^ (*p* is the degree of polynomial) and $K_{Gaussian}(x_{i},x_{j})=e^{\frac{{\left\|x_{i}-x_{j}\right\|}^{2}}{2\sigma }}$ (*σ* is Gaussian sigma), respectively.

Related works have shown that there is no formal way to decide the best kernel function for a specific domain problem. However, among different kernel functions, linear, polynomial, and radial basis function kernels are the most widely used and compared in various domain problems, such as crop classification [22], gene expression classification [23], protein localization [24], speaker identification [25], and splice site identification [26].

### Classifier Ensembles

Classifier ensembles combining multiple classifiers has come to be regarded as an important pattern classification technique [27–29] offering improved classification performance of a single classifier [16].

The concept behind classifier ensembles is inspired by the nature of information processing in the brain, which is modular. That is, individual functions can be subdivided into functionally different sub-process or subtasks without mutual interference [30]. This is the divide-and-conquer principle that enables a complex problem to be divided into simpler sub-problems (i.e., simpler tasks), which can then be solved with different learning methods or algorithms.

Two widely used techniques for combining multiple classifiers are bagging and boosting. In bagging, several classifiers are trained independently by different training sets via the bootstrap method [31]. Bootstrapping builds *k* replicate training data sets to construct *k* independent classifiers by randomly re-sampling the original given training dataset, but with replacement. That is, each training example may appear to be repeated in any in any particular replicate training data set of *k*, or not at all. Then, the *k* classifiers are aggregated via an appropriate combination method, such as majority voting [32].

In boosting, like bagging, each classifier is trained using a different training set. However, the *k* classifiers are trained not in parallel and independently, but sequentially. The original boosting approach, *boosting by filtering*, was proposed by Schapire [33]. Nowadays, AdaBoost (or Adaptive Boosting) is the most common boosting learning algorithm used in pattern recognition.

Initially, each example of a given training set has the same weight. For training the *k*-th classifier as a *weak learning model*, *n* sets of training samples (*n* < *m*) among *S* are used to train the *k*-th classifier. Then, the trained classifier is evaluated by *S* to identify those training examples which cannot be classified correctly. The *k* + 1 classifier is then trained by a modified training set which boosts the importance of those incorrectly classified examples. This sampling procedure will be repeated until *K* training samples are built for constructing the *K-*th classifier. The final decision is based on the weighted vote of the individual classifiers [34].

### Comparisons of Related Works using SVM

Table 1 compares several recent related works on breast cancer prediction using SVM in terms of the kernel function employed, the dataset used, and evaluation metric considered. Note that some studies use the Weka data mining software (available at: http://www.cs.waikato.ac.nz/ml/weka/) to construct the SVM classifier and they do not specify the kernel function used. In this case, we assume that they consider the default parameters of SVM, i.e., the RBF kernel function.

**Table 1:** Comparison of related works.

Work	Kernel function	Dataset resource	Evaluation metric
Joshi et al. (2014) [35]	RBF	Breast Cancer Wisconsin	Accuracy
Senturk and Kara (2014) [14]	—	Breast Cancer Wisconsin	Accuracy; Sensitivity; Specificity
Ahmad et al. (2013) [11]	RBF	The Iranian Center for Breast Cancer dataset	Accuracy; Sensitivity; Specificity
Salama et al. (2012) [13]	RBF	Breast Cancer Wisconsin	Accuracy
Aruna et al. (2011) [3]	RBF	Breast Cancer Wisconsin	Accuracy; Sensitivity; Specificity
Abdelaal et al. (2010) [10]	RBF	Digital Database for Screening Mammography	ROC curve
Rong and Yuan (2010) [36]	RBF	Breast Cancer Wisconsin	Accuracy
You and Rumbe (2010) [15]	Poly/RBF/Sigmoid	Breast Cancer Wisconsin	Accuracy
Huang et al. (2008) [12]	RBF	Chung-Shan Medical University Hospital	Accuracy

From Table 1, several limitations of these recent studies can be identified. With the exception of You and Rumbe [15], most related works constructing the SVM classifier for breast cancer prediction are only based on the RBF kernel function. Although RBF is the most widely used kernel function in SVM, the prediction performance obtained using other different popular kernel functions has not yet been fully explored. Second, most studies have only used the Breast Cancer Wisconsin dataset in their experiments. Although this is a publicly available dataset (available at: https://archive.ics.uci.edu/ml/datasets/Breast+Cancer+Wisconsin+(Original)), its dataset size is too small to effectively validate the performance of SVM for breast cancer prediction. Therefore, in this paper, another large scale breast cancer dataset will also be used for further comparison.

Finally, the prediction (or classification) accuracy is usually the primary evaluation metric used to assess the performance of prediction models. However, the collected dataset for breast cancer prediction is usually classified as a class imbalance problem. That is, the class for the patients having the cancer contains a very small number of data samples whereas the class for the normal patients without cancer contains a very large number of data samples. This causes a problem when examining the accuracy of the prediction model because the error of incorrectly classifying a normal patient without cancer as part of the cancer class and the error of incorrectly classifying a patient having cancer as part of the normal class are not assessed. Only half of the related works shown in Table 1 consider both the sensitivity (i.e., the true positive rate) and specificity (i.e., the false positive rate) indexes or the receiver operating characteristic (ROC) curve, which is based on sensitivity and specificity together. Therefore, in addition to the classification accuracy, the ROC curve, the F-measure rate or the F-1 score [37] will also be examined in this study. The F-measure considers both the precision (the number of correct positive results divided by the number of all positive results) and recall (the number of correct positive results divided by the number of positive results that should have been returned) when computing the score. In other words, it is a weighted average of the precision and recall.

## Experimental Methodology

### Experimental Procedure

The experimental procedure is based on the following steps. First of all, the given dataset is divided into 90% training and 10% testing sets based on the 10-fold cross validation strategy [38]. In the second step the focus is on constructing the SVM classifiers using different kernel functions (i.e., linear, polynomial, and RBF) individually. Moreover, SVM classifier ensembles will also be constructed by bagging and boosting to produce linear, polynomial, and RBF SVM ensembles. Finally, the testing set is fed into the constructed classifiers prior to examination of their classification accuracy, ROC, and F-measure rates. Furthermore, the classifier training times are also compared to analyse the computational complexities of training different classifiers.

We also examine whether performing feature selection to filter out unrepresentative features from the chosen dataset can make the classifiers perform better than the ones without feature selection. In this case, the genetic algorithm (GA) [39] is used.

### Experimental Setup

#### The Datasets

In this paper, two breast cancer datasets are used, which are available from the UCI machine learning repository (available at: http://archive.ics.uci.edu/ml/) and ACM SIGKDD Cup 2008 (available at: http://www.sigkdd.org/kddcup/index.php). The former is a relatively small scale dataset, which is composed of 699 data samples and each data sample has 11 different features. On the other hand, the latter dataset contains 102294 data samples and each data sample is represented by 117 different features, which is regarded as a large scale dataset in this paper.

#### The Classifier Design

To construct different SVM classifiers, the Weka data mining software is used. Besides the kernel functions which are chosen for developing specific SVM classifiers, other related parameters are based on the default values of Weka. The same approach is used for constructing SVM ensembles based on bagging and boosting.

Consequently, there are three single SVM classifiers, namely, linear SVM, polynomial SVM, and RBF SVM, and six SVM ensembles, namely, linear/polynomial/RBF SVM ensembles constructed by bagging and boosting, respectively. In addition, to evaluate the performance of the different SVM classifiers, in addition to the classification accuracy, ROC, and the F-measure rate, the time that is spent training each classifier is also compared. Note that the computing environment is based on PC, Intel^®^ Core^™^ i7-2600 CPU @ 3.40GHz, 4 GB RAM.

We also use Weka to perform the task of feature selection using the genetic algorithm and its parameters are based on the default values.

## Experimental Results

### Single SVM Classifiers

**Fig 2:**
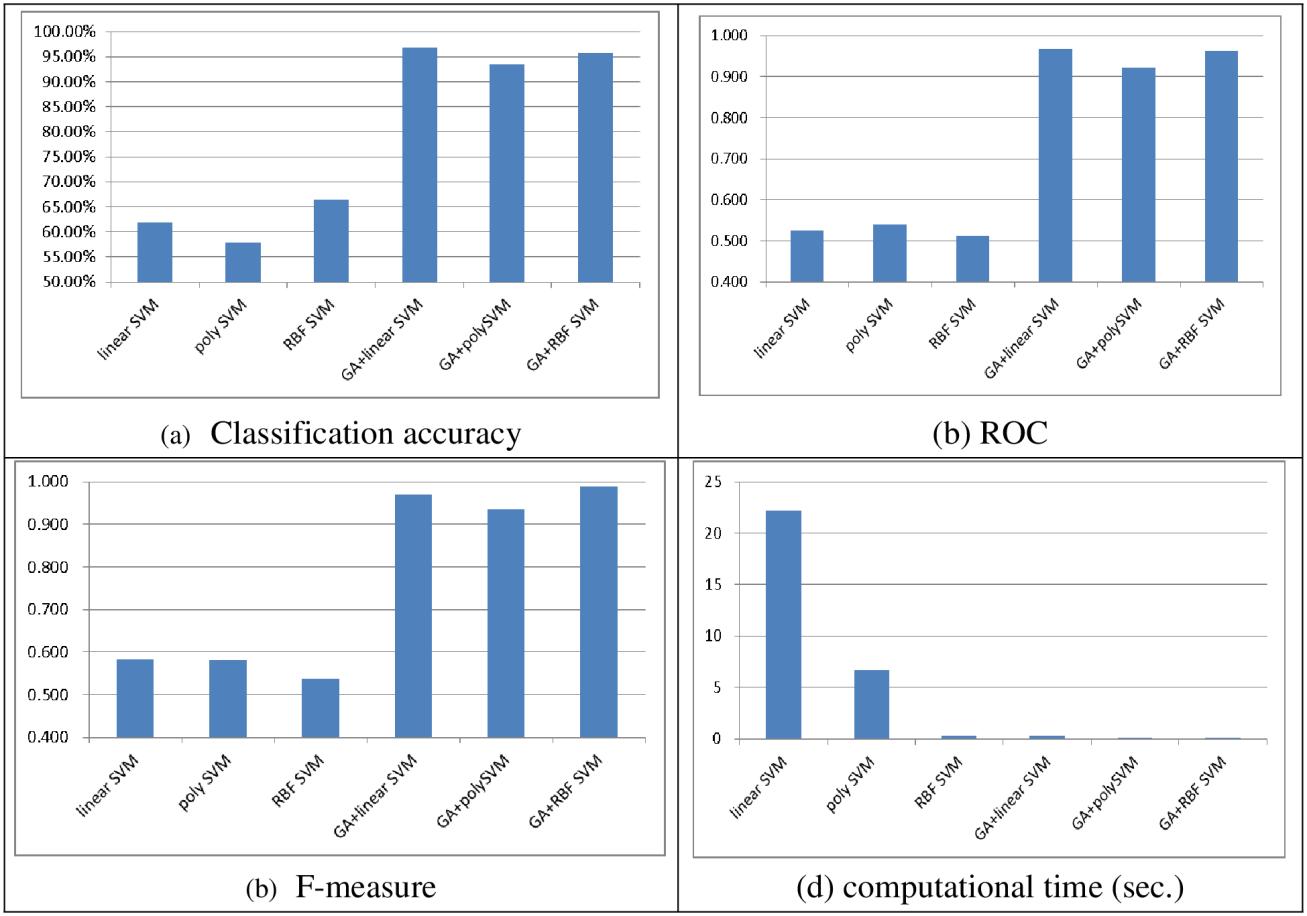
The performance of single SVM classifiers over the small scale dataset. (A) Classification accuracy, (B) ROC, (C) F-measure, (D) Computational time (sec.)

**Fig 3:**
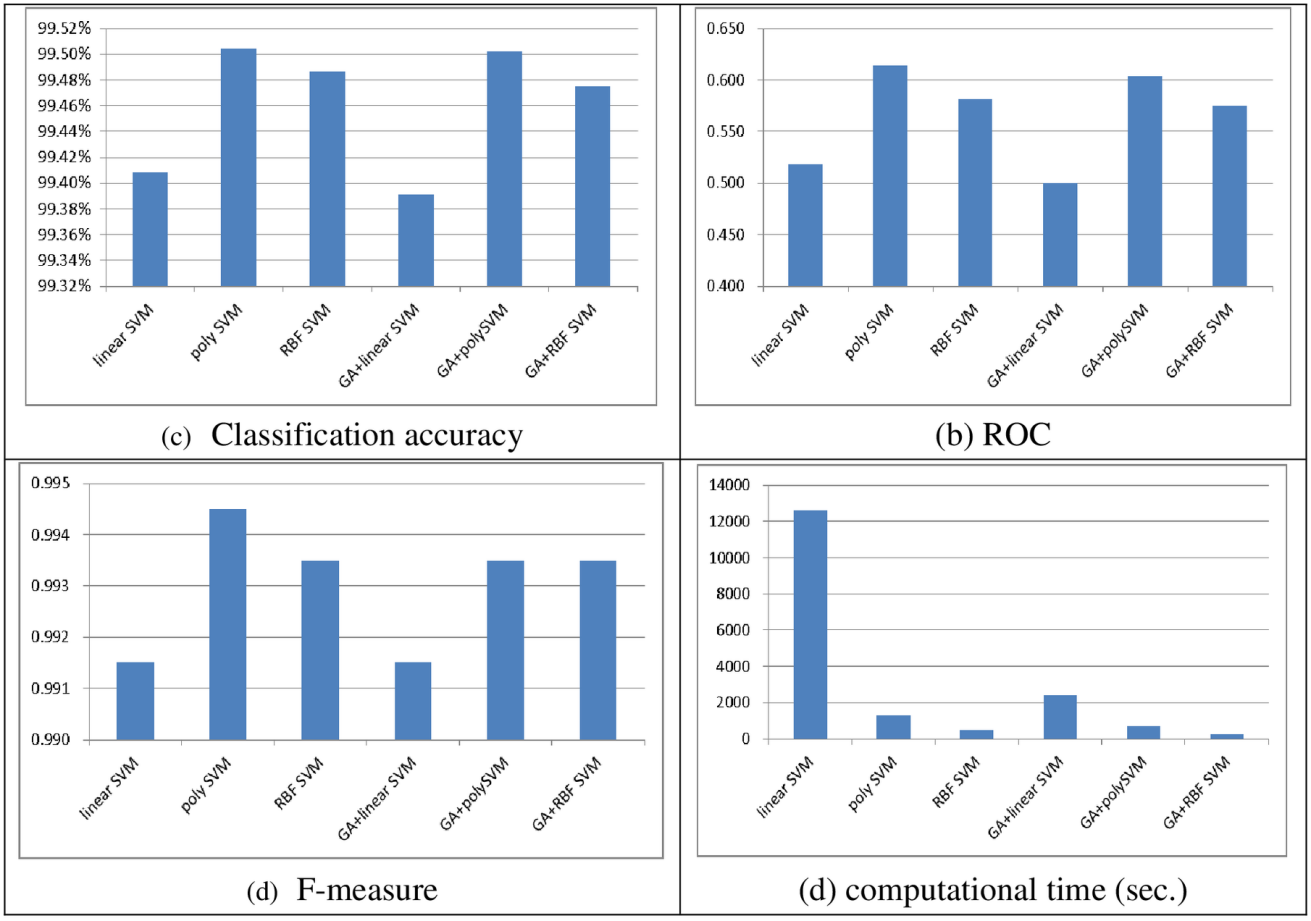
The performance of single SVM classifiers over the large scale dataset. (A) Classification accuracy, (B) ROC, (C) F-measure, (D) Computational time (sec.)

Figs 2 and 3 show the performances of the SVM classifiers obtained with linear, polynomial, and RBF kernel functions with and without feature selection in terms of classification accuracy, ROC, the F-measure, and the computational time (in seconds) over the two datasets, respectively. Note that after performing feature selection using the genetic algorithm, the numbers of features selected from the small and large scale datasets are 10 and 36, respectively.

As we can see, performing feature selection before training the SVM classifiers allows them to produce significantly better performance (i.e., classification accuracy, ROC, and F-measure). In particular, the best performances are obtained by GA + linear SVM for classification accuracy (96.85%), GA + linear SVM for ROC (0.967), and GA + RBF SVM for F-measure (0.988). Moreover, there are no big performance differences between GA + linear SVM and GA + RBF SVM.

In addition, there is a large reduction in the computational times for training the SVM classifiers after performing feature selection compared with the baseline SVM classifiers without feature selection. Comparison of the training times shows that training RBF SVM requires the least time and poly SVM requires the second smallest time. The largest computational time is required for the linear SVM classifier.

With a large scale dataset, performing feature selection does not necessarily make the SVM classifiers perform better than the ones without feature selection. Specifically, the best performances are obtained by poly SVM and GA + poly SVM for classification accuracy (99.50%) and poly SVM for ROC (0.614) and F-measure (0.994). Similar to the results obtained with the small scale dataset, the SVM classifiers with and without feature selection based on the polynomial and RBF kernel functions perform similarly in terms of classification accuracy and F-measure, specifically, the performance differences are 0.02% for classification accuracy and 0.001 for the F-measure. However, when the ROC is considered as the evaluation metric, poly SVM significantly outperforms the other SVM classifiers.

Comparison of the computational times for training the SVM classifiers shows that the largest computational time is required for the linear SVM classifier without feature selection. After performing feature selection the training times for the other classifiers, i.e., poly SVM and RBF SVM are about twice as small as the ones without feature selection. However, from our perspective, there is not a big difference between the 11 minutes spent by GA + poly SVM and 20 minutes spent by poly SVM, especially when the latter prediction model produces the best performance in terms of classification accuracy, ROC, and F-measure.

In short, GA + RBF SVM and poly SVM are better choices for the small and large scale datasets, respectively, since they can provide better performance in terms of classification accuracy, ROC, and F-measure, and they do not require large classifier training times.

### SVM Classifier Ensembles

**Fig 4:**
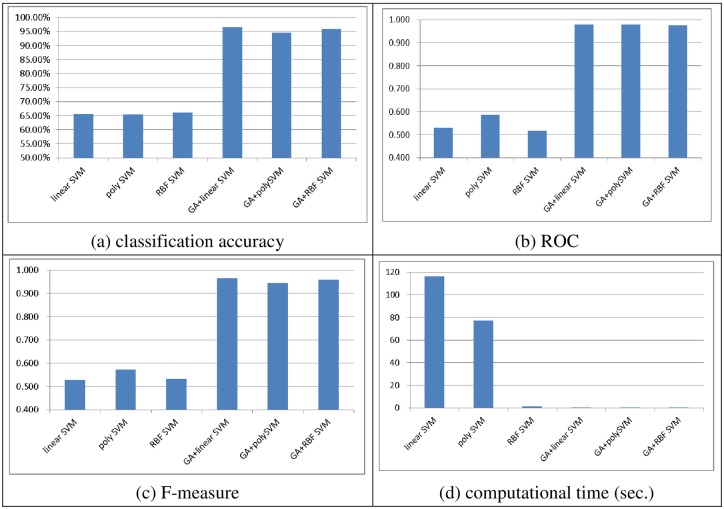
The performance of the bagging based SVM classifier ensembles over the small scale dataset. (A) Classification accuracy, (B) ROC, (C) F-measure, (D) Computational time (sec.)

**Fig 5:**
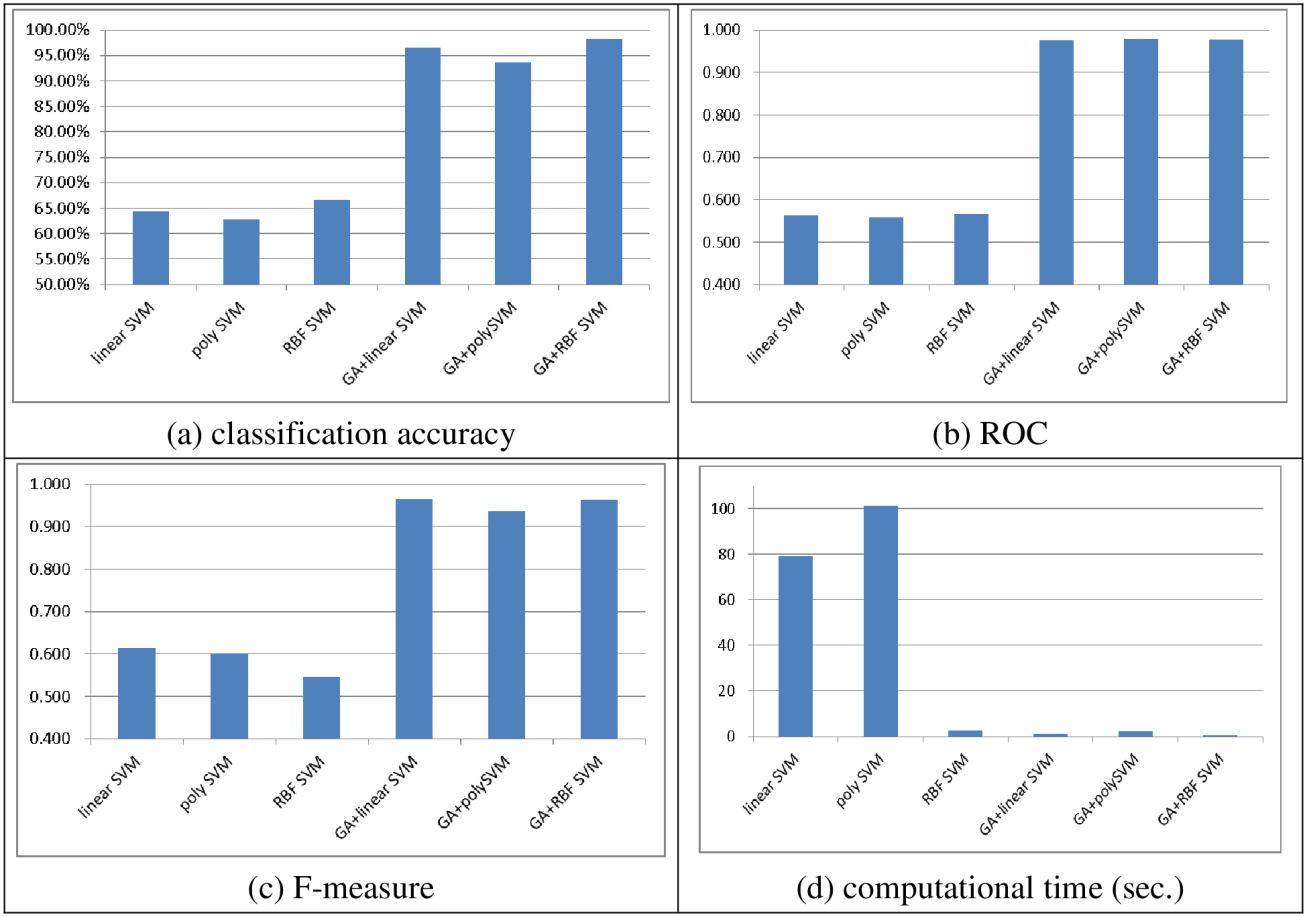
The performance of the boosting based SVM classifier ensembles over the small scale dataset. (A) Classification accuracy, (B) ROC, (C) F-measure, (D) Computational time (sec.)

**Fig 6:**
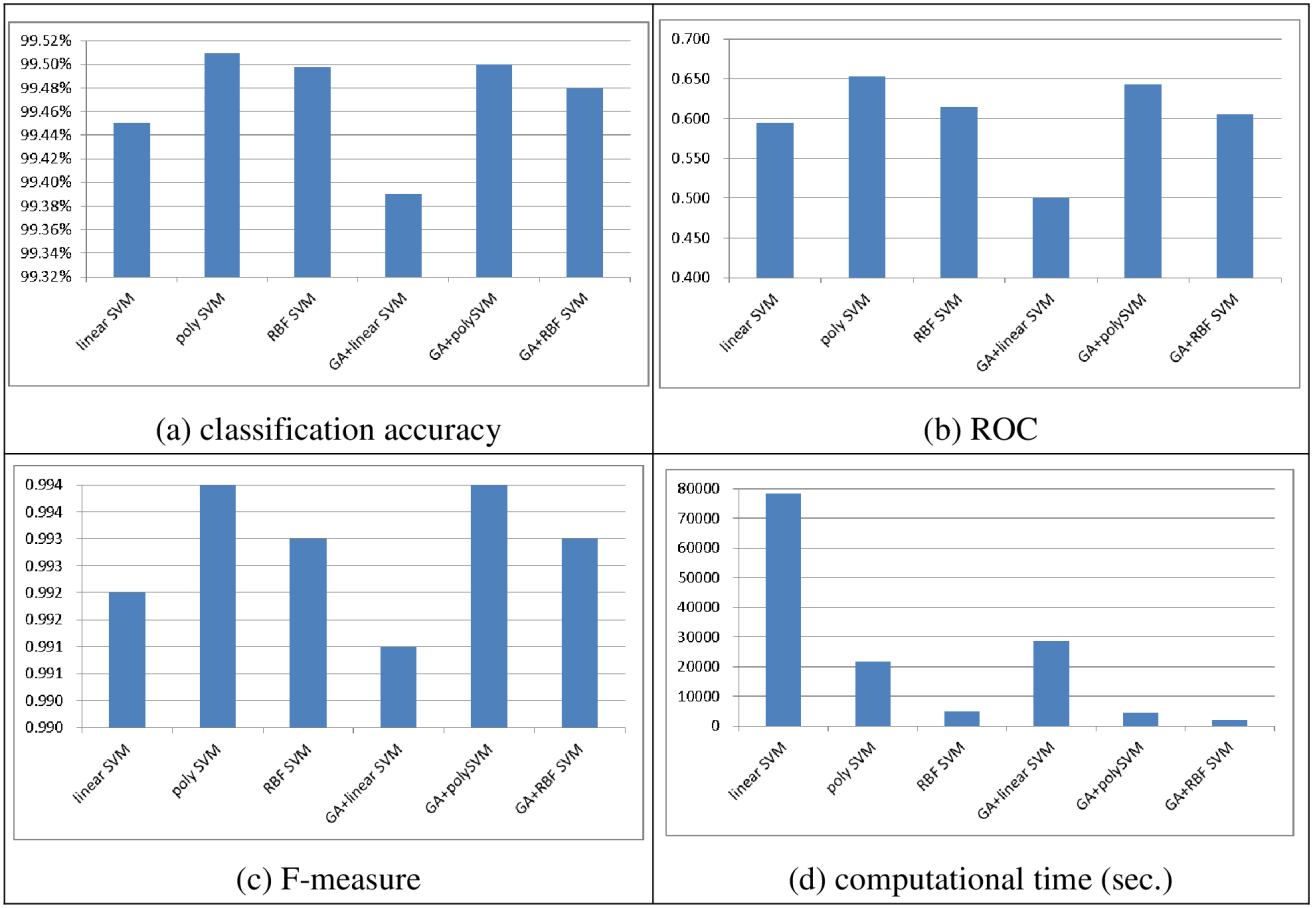
The performance of the bagging based SVM classifier ensembles over the large scale dataset. (A) Cla.ssification accuracy, (B) ROC, (C) F-measure, (D) Computational time (sec.)

**Fig 7:**
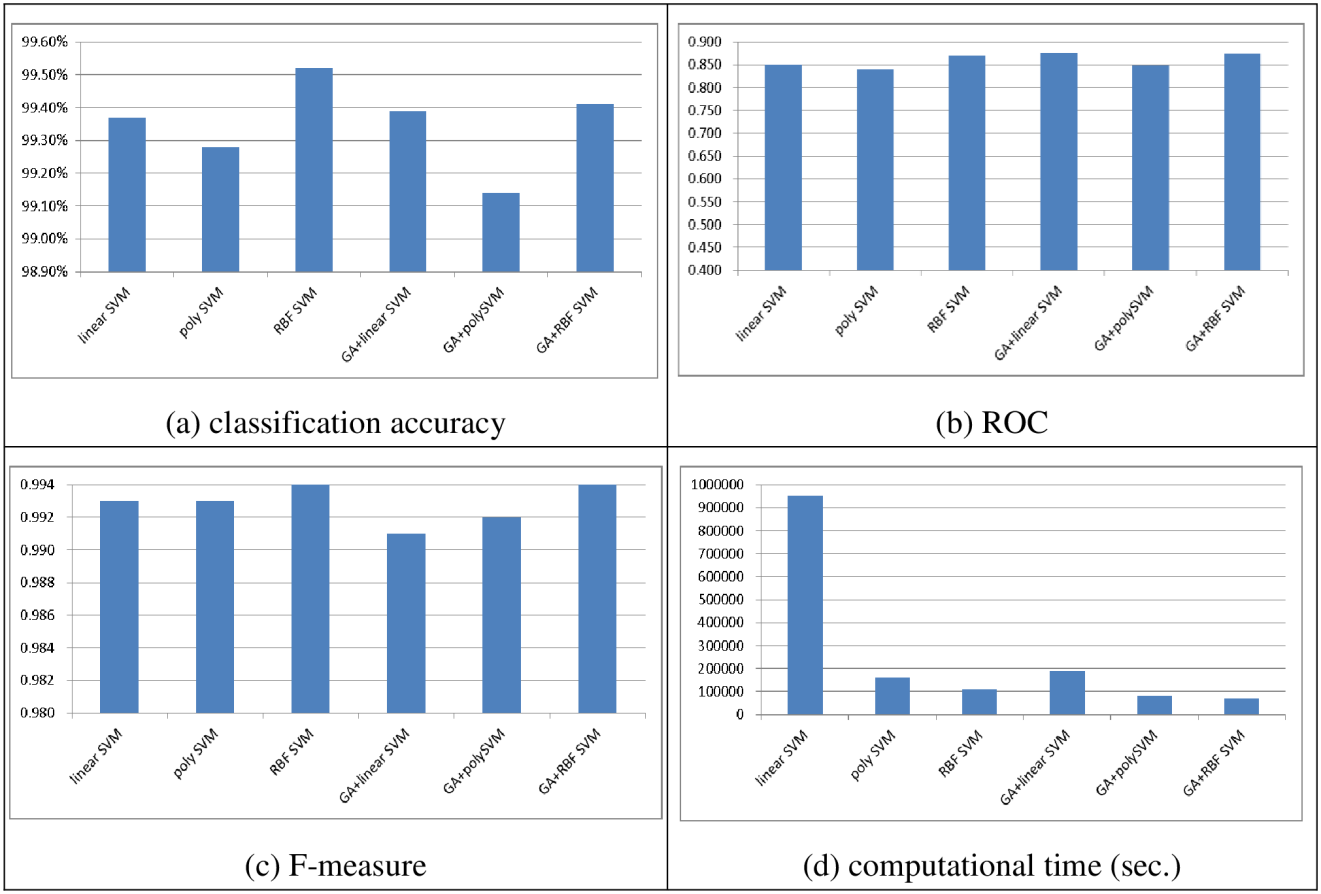
The performance of the boosting based SVM classifier ensembles over the large scale dataset. (A) Classification accuracy, (B) ROC, (C) F-measure, (D) Computational time (sec.)

Figs 4 to 7 show the performances of linear, poly, and RBF SVM classifier ensembles obtained by bagging and boosting with and without feature selection, in terms of the classification accuracy, ROC, F-measure, and computational time, over the two datasets, respectively.

For the small scale dataset, similar to single SVM classifiers (c.f., Fig 2(a) to 2(c)), combining GA with SVM ensembles outperforms SVM ensembles without feature selection no matter which kernel function and ensemble method are used. In particular, the GA + RBF SVM ensembles using the boosting method perform the best in terms of classification accuracy (98.28%), while the GA + linear SVM ensembles and GA + poly SVM ensembles using the bagging method outperform the other classifier ensembles (0.98), and the GA + linear SVM using both the bagging and boosting methods can provide the highest F-measure rate (0.966).

Training the SVM ensembles based on the reduced dataset after performing GA leads to a large reduction in the computational time. In particular, the RBF SVM ensembles require less training time than the linear and poly SVM ensembles when the original dataset is used. This result is similar to the previous one (c.f. Fig 2(d)).

With a large scale dataset, performing feature selection does not make the SVM ensembles perform better than without feature selection. Specifically, the best performance is obtained using the RBF SVM ensembles and the boosting method, GA + linear SVM ensembles and the boosting method, and RBF SVM ensembles and the boosting method in terms of classification accuracy (99.52%), ROC (0.876), and F-measure (0.995).

The SVM ensembles using the boosting method require larger training times than the ones using the bagging method. However, RBF SVM ensembles require less time than the other SVM ensembles.

### Discussion

There is no single classifier that can perform the best for all of the evaluation metrics. Table 2 lists the top 3 classifiers based on the classification accuracy, ROC, and F-measure for further comparison.

**Table 2:** Comparison of the the classification accuracy, ROC, and F-measure of the top 3 classifiers.

Classification accuracy		ROC		F-measure	
*Small scale dataset*					
1	GA+RBF SVM ensembles (boosting) (98.28%)	1	GA+linear/poly SVM ensembles (bagging) (0.98)	1	GA+RBF SVM (0.988)
2	GA+linear SVM (96.85%)	2	GA+poly SVM ensembles (boosting) (0.979)	2	GA+linear SVM ensembles (bagging/boosting) (0.966)
3	GA+linear SVM ensembles (bagging/boosting) (96.57%)	3	GA+RBF SVM ensembles (boosting) (0.977)	3	GA+RBF SVM ensembles (boosting) (0.963)
*Large scale dataset*					
1	RBF SVM ensembles (boosting) (99.52%)	1	GA+linear SVM ensembles (boosting) (0.876)	1	RBF SVM ensembles (boosting) (0.995)
2	Poly SVM ensembles (bagging) (99.51%)	2	GA+RBF SVM ensembles (boosting) (0.875)	2	Poly SVM; poly SVM ensembles (bagging); GA+poly SVM ensembles (bagging); GA+RBF SVM ensembles (boosting) (0.994)
3	Poly SVM; GA+poly SVM; RBF SVM ensembles (bagging); GA+poly SVM ensembles (bagging) (99.50%)	3	RBF SVM ensembles (boosting) (0.869)		

We can observe that SVM ensembles mostly provide better performances than single SVM classifiers. This finding is consistent with those in related studies (Kittler et al., 1998). In addition, for the small scale dataset, GA + linear SVM ensembles by bagging and GA + RBF SVM ensembles by boosting can be regarded as better classifiers for different evaluation metrics. On the other hand, for the large scale dataset, only RBF + SVM ensembles by boosting are in the top 3 lists of the three different evaluation metrics.

When the computational time is also compared, the training time of the GA + linear SVM with bagging is similar to the one for the GA + RBF SVM ensembles with boosting when a small scale dataset is used (i.e., 0.57 vs. 0.5). For the better classifier over the large scale dataset, the RBF SVM ensembles with boosting require about 301 hours, which is smaller than the average required by SVM ensembles with boosting (724 hours), but higher than the average for SVM ensembles with bagging (65 hours).

Therefore, when a larger dataset is used, and the prediction performances and the classifier training time are both considered simultaneously, we recommend the GA + RBF SVM ensembles based on boosting. This is because they provide a classification accuracy, ROC, and F-measure of 99.41%, 0.875, and 0.994, respectively. In addition, they require about 186 hours, which is much less than for the RBF SVM ensembles with boosting. However, if a cloud platform is used, such as with the MapReduce computation implemented using Hadoop (available at: https://hadoop.apache.org/), the computational burden can be certainly lessened. In this case, RBF SVM ensembles with boosting are the optimal choice for the breast cancer prediction model.

It should be noted that these findings are only suitable for the breast cancer prediction datasets. That is, these two datasets contain small numbers of features and large numbers of data samples, i.e. 11 vs. 699 for the small scale dataset and 117 vs. 102294 for the large scale dataset. Here, two additional datasets that contain very large numbers of features but smaller numbers of data samples are used for further analyses, i.e. the numbers of features are larger than the numbers of data samples. They are the Arcene (available at: https://archive.ics.uci.edu/ml/datasets/Arcene) and MicroMass (available at: https://archive.ics.uci.edu/ml/datasets/MicroMass) datasets, which contain 10000 features vs. 900 data samples and 1300 features vs. 931 data samples, respectively.

Table 3 shows the results of different SVM classifiers using the two datasets. As we can see that the RBF SVM classifiers perform the worst over this kind of datasets. On the other hand, for the linear SVM and poly SVM classifiers, constructing classifier ensembles by the bagging and boosting methods do not always outperform single classifiers. Particularly, these results indicate that using the single linear SVM classifier is a good baseline classifier for the datasets that contain very large numbers of features, which are larger than the numbers of data samples.

**Table 3:** Classification accuracies of single SVM and SVM ensembles over the Arcene and MicroMass datastes.

	Linear SVM	Poly SVM	RBF SVM
*Arcene dataset*			
Single	0.885	0.89	0.56
Bagging	0.9	0.88	0.56
Boosting	0.89	0.895	0.56
*MicroMass dataset*			
Single	0.786	0.648	0.105
Bagging	0.772	0.639	0.105
Boosting	0.769	0.624	0.105

## Conclusion

In this paper, the performance of single SVM classifiers and SVM classifier ensembles obtained by using different kernel functions and different combination methods are examined in terms of breast cancer prediction. In addition, two different scaled datasets are used for comparison. Moreover, classification accuracy, ROC, F-measure, and the computational time of training different classifiers are compared.

These specific experimental settings have never been shown before and the experimental results allow us to fully understand the prediction performances of SVM and SVM ensembles and the better prediction model(s) can be identified as the baseline classifier(s) for future studies.

We found that most SVM ensembles performed slightly better than single SVM classifiers. In particular, performing feature selection using the genetic algorithm (GA) over the small scale dataset can make single SVM classifiers and SVM ensembles provide significantly better performances than the same classifiers without feature selection. Among them, the GA + linear SVM ensembles with bagging and GA + RBF SVM ensembles with boosting are the top two prediction models and their performance differences are not significant.

On the other hand, for the large scale dataset, a prediction model based on RBF SVM ensembles with boosting is the better choice. However, SVM ensembles based on boosting usually take a longer training time than single SVM classifiers and SVM ensembles with bagging. In practice, there are two possible solutions to lower the computational time. The first one is to perform feature selection first to reduce the dataset dimensionality. In this case, GA + SVM ensembles based on boosting still provide better performances than many other classifiers. The second one is to directly construct SVM ensembles by boosting taking advantage of a cloud platform. In this case, there is no need to perform feature selection, while the classifier training time can still be greatly reduced.
